# Carbapenem-Resistant *Acinetobacter baumannii* in Zagreb, Croatia, in Post-COVID-19 Pandemic Period: Resistance Trends and Mechanisms

**DOI:** 10.3390/microorganisms14051123

**Published:** 2026-05-15

**Authors:** Branka Bedenić, Marina Nađ, Vesna Bratić, Daniela Bandić Pavlović, Mislav Kasalo, Mirela Dobrić, Rocío Arazo del Pino, Tessa Burgwinkel, Andrea Grisold, Josefa Luxner, Gernot Zarfel, Paul G. Higgins

**Affiliations:** 1 Department of Medical Microbiology and Parasitology, University of Zagreb School of Medicine, 10000 Zagreb, Croatia; 2 Clinical Department for Clinical Microbiology, Infection Control and Prevention, University Hospital Center Zagreb, University of Zagreb, 10000 Zagreb, Croatia; 3 University of Zagreb School of Medicine, 10000 Zagreb, Croatia; mnad@student.mef.hr; 4 Department of Anesthesiology and Intensive Care, University Hospital Centre Zagreb, 10000 Zagreb, Croatia; vbratic@kbc-zagreb.hr (V.B.); daniela.bandic.pavlovic@mef.hr (D.B.P.); kasalomislav@gmail.com (M.K.); 5 Department of Anesthesiology and Intensive Care, University of Zagreb School of Medicine, 10000 Zagreb, Croatia; 6 Department of Anesthesiology, Intensive Medicine and Pain Management, University Hospital Centre Sestre Milosrdnice, 10000 Zagreb, Croatia; dobric.mirela@gmail.com; 7 Institute for Medical Microbiology, Immunology and Hygiene, Faculty of Medicine and University Hospital Cologne, University of Cologne, 50923 Cologne, Germany; rocio.arazo-del-pino@uk-koeln.de (R.A.d.P.); tessa.burgwinkel@uk-koeln.de (T.B.); 8 Diagnostic and Research Institute for Hygiene, Microbiology and Environmental Medicine, Medical University of Graz, 8010 Graz, Austria; andrea.grisold@medunigraz.at (A.G.); josefa.luxner@medunigraz.at (J.L.); gernot.zarfel@medunigraz.at (G.Z.); 9 German Center for Infection Research (DZIF), Partner Site Bonn-Cologne, 50931 Cologne, Germany; paul.higgins@uni-koeln.de

**Keywords:** carbapenem-resistant *Acinetobacter baumannii*, carbapenem-hydrolyzing oxacillinases, OXA-23-like, OXA-24/40-like

## Abstract

During the COVID-19 pandemic carbapenem-resistant *Acinetobacter baumannii* (CRAB) was found to be the major pathogen associated with ventilator-associated pneumonia in mechanically ventilated patients. This prompted us to analyze the post-pandemic mechanisms of carbapenem resistance, antibiotic resistance trends, and molecular epidemiology of CRAB in Croatia. In total, 94 CRAB isolates from two hospital centers, including outpatient settings, were investigated. Antimicrobial susceptibility testing was performed by broth microdilution. PCR was used to detect genes encoding carbapenemases of group A, B and D and extended-spectrum β-lactamases (ESBL). Randomly selected isolates were subjected to whole resistome analysis by Inter-array CarbaResist Kit and whole-genome sequencing (WGS). Phylogenetic tree and sequence types (STs) were retrieved from WGS. Plasmid incompatibility groups were determined by PCR-based replicon typing (PBRT). All isolates were extensively drug resistant (XDR), showing resistance to ceftazidime, cefepime, piperacillin–tazobactam, imipenem, meropenem, gentamicin, amikacin and ciprofloxacin, and 13% (n = 12) were also resistant to colistin. The Hodge and CIM test exhibited poor sensitivity with only 32 and 30% of isolates being identified as carbapenemase producers, respectively. PCR identified *bla*_OXA-23_ as the dominant carbapenemase gene in both hospitals, found in 71% of the isolates (67/94). In an outpatient setting, *bla*_OXA-24/40_ was dominant. *bla*_OXA-23_ and *bla*_OXA-72_ were the only allelic variants. The Inter-array CarbaResist Kit and whole-genome sequencing (WGS) identified a variety of aminoglycoside (*armA*, *ant(3″)-IIa*, *aph(3″)-Ib*, *aph(6)-Id*) and sulphonamide resistance (*sul1* and *sul2*) genes. The representative *bla*_OXA-23_-positive isolates belonged to ST2, while *bla*_OXA-72_-positive isolates were allocated to ST492. These data show that there are different populations of XDR *A. baumannii* between hospital and outpatients.

## 1. Introduction

*Acinetobacter baumannii*, an opportunistic pathogen belonging to the ESKAPE group, has emerged as a formidable agent of nosocomial infections worldwide [1,2].

As an opportunistic pathogen, it is associated with nosocomial infections, including ventilator-associated pneumonia (VAP), bloodstream infections (BSI), wound infections, and meningitis [2,3]. Its amazing capacity to acquire antibiotic resistance determinants from other Gram-negative bacteria has led to an extensively drug-resistant phenotype (XDR). Carbapenem resistance of this superbug is primarily due to the production of carbapenemases, mostly belonging to Ambler class D oxacillinases (OXA), also known as carbapenem hydrolyzing oxacillinases (CHDL). OXA-23-like, OXA-24/40-like and OXA-58-like are the most widespread CHDL’s in *A. baumannii* [4]. Other subgroups, OXA-143-like and OXA-235-like are rarer. Class B carbapenemases (NDM, VIM, IMP, SIM), zinc-dependent integron-borne metallo-β-lactamases (MBLs), are less common, while class A (KPC, GES) enzymes, widespread among *Enterobacterales,* are rare among *A. baumannii* [4]. Overproduction of chromosomal AmpC β-lactamase, loss of CarO porin and upregulation of efflux pumps are reported to contribute to carbapenem resistance [5]. Increased resistance to cephalosporins may also be due to the intrinsic cephalosporinase, or acquired extended-spectrum β-lactamases (ESBLs). ESBLs are class A β-lactamases, mostly plasmid-mediated enzymes that are able to hydrolyze third- and fourth-generation cephalosporins and monobactams. The ESBLs in *A. baumannii* include TEM, SHV, GES, PER and VEB [6]. Previous studies in Croatia have identified OXA-24/40-like as the dominant CHDL among carbapenem-resistant *A. baumannii* at the beginning of the third millennium, with OXA-72 as the only allelic variant [7,8], but later it was outnumbered by OXA-23-like, which is now the dominant CHDL in Croatia and elsewhere in Europe [9]. OXA-23 was found to be the most frequent CHDL in both a hospital setting [9] and nursing homes [10]. *bla*_OXA-23_ genes were found to be associated with the IS*Aba1* insertion sequence upstream of the gene. Molecular epidemiology analysis has revealed that the majority of Croatian isolates belonged to international clonal lineage (IC) 2 [7,8].

*A. baumannii* high-risk clones (International Clones (1–12) are globally disseminated multidrug-resistant lineages that cause severe nosocomial infections, with IC2 (ST2/Global Clone 2) being the most prevalent and dominant in intensive- care units (ICUs) worldwide. These clones, particularly ST2 and the emerging ST697, carry extensive resistance genes (*bla*_OXA-23_, *arm*A) and pose a severe threat due to their rapid evolution, high virulence, and environmental persistence [11].

During the COVID-19 pandemic, carbapenem-resistant *A. baumannii* (CRAB) established itself as the most frequent pathogen associated with VAP in mechanically ventilated COVID-19 patients in Croatia [12,13] and other geographic regions, causing a poor outcome due to an XDR phenotype and limited therapeutic options. Furthermore, in the past, CRAB was confined to the hospital and nursing home niches, but lately, during routine diagnostics, we have observed CRAB increasingly in the outpatient setting. Thus, the aim of this study was to analyze and compare carbapenemases in CRAB isolated from the hospital and outpatient settings in Zagreb, Croatia, and to analyze their resistance trends post-COVID-19 pandemic. Moreover, the susceptibility to the novel last-line antimicrobial compounds such as cefiderocol were also investigated, and the clinical outcomes were investigated.

## 2. Materials and Methods

### 2.1. Bacterial Isolates and Patients

Consecutive non-duplicate (one per patient) *A. baumannii* isolates were collected from two hospital centers in Zagreb (University Hospital Centre Zagreb (UHCZ) and University Hospital Centre Sestre Milosrdnice (UHCSM)), including the outpatient setting belonging to UHCZ from 1 June 2022 to 3 July 2025. The isolates were identified to the species level using MALDI-TOF and confirmed as *A. baumannii* by PCR for *bla*_OXA-51_ genes. Patient data, including age, gender, type of infection, comorbidities, length of hospital stay and therapeutic outcome, were retrieved from the hospital internet system (bis) for hospitalized patients (n = 84). The Charlston comorbidity index (CCI) was calculated. A CCI value of 0 means that there are no comorbidities, 1–2 indicates low risk, 3–4 moderate risk, and >4 high risk.

### 2.2. Antimicrobial Susceptibility Testing and Phenotypic Tests for Detection of Extended-Spectrum β-Lactamases and Carbapenemases

The antimicrobial susceptibility of *A. baumannii* isolates was investigated by Kirby–Bauer standard disk diffusion test (DDST) according to the European Committee on Antimicrobial Susceptibility testing (EUCAST) [14] for the following antibiotics: ceftazidime (30 μg) cefepime (30 μg), imipenem (10 μg), meropenem (10 μg), gentamicin (10 μg), amikacin (30 μg), ciprofloxacin (5 μg), levofloxacin (5 μg), sulphamethoxazole–trimethoprim (23.75 + 1.25 μg) and cefiderocol (30 μg). For piperacillin–tazobactam (110 μg) and ampicillin–sulbactam (30 μg) CLSI guidelines were used, as there are no breakpoints provided by EUCAST. The disks were purchased from Oxoid, Basingstoke, UK. The broth microdilution methods, in line with the Clinical and Laboratory Standard Institute (CLSI) [15], was applied to confirm resistance to ceftazidime, cefepime, imipenem, meropenem, gentamicin, amikacin and ciprofloxacin. The interpretive measures of the CLSI were followed to categorize *Acinetobacter* isolates as resistant, intermediate susceptible, or susceptible [15]. Colistin susceptibility testing was done by broth dilution method and interpreted according to EUCAST guidelines since there are no guidelines provided by CLSI [14]. *A. baumannii* ATCC 19606 was utilized as the standard quality control strain. MIC_90_ and MIC_50_ of the tested antimicrobials were determined as the lowest concentrations, which inhibit 90% and 50% of the tested isolates. The strains were defined as multidrug resistant (MDR), extensively drug resistant (XDR) or pandrug resistant (PDR), according to Magiorakos et al. [16]. MICs of imipenem and meropenem were tested also in the presence of cloxacillin to determine the effect of chromosomal AmpC overproduction on the carbapenem resistance [17].

The modified Hodge test (MHT) and m CIM test were used to assess carbapenem hydrolysis. Two methods were applied due to low sensitivity of phenotypic tests in *A. baumannii*. MHT was performed according to the CLSI 2017 (M100-S31) document [18]. Overnight culture of the carbapenem-susceptible indicator strain *Escherichia coli* ATCC 25922 was inoculated on the surface of Mueller–Hinton agar (MHA) plates. After drying, an ertapenem disk (10 µg) was placed in the middle of the plate. Overnight *A. baumannii* cultures were streaked as a single line from the periphery of the disk to the edge of the plate. The positive result showed a clover-leaf-shaped zone of inhibition of *E. coli* ATCC 25922 growth along the test *A. baumannii* growth streak within the disk diffusion zone. The negative result showed no growth of *E. coli* ATCC 25922 along the test *A. baumannii* growth streak within the diffusion zone. Positive control strains producing various types of carbapenemases were from our own collection.

The mCIM and m/eCIM tests were performed according to the Clinical and Laboratory Standards Institute, 2021 guidelines [19]. The mCIM was performed for all the isolates, whereas the eCIM (in conjunction with mCIM) was performed on isolates that initially tested positive for carbapenemases in the mCIM test, as suggested by the CLSI. The interpretation of the test positivity was based on the inhibition zone of *E. coli* ATCC 25922 in mCIM and eCIM [19]. Briefly, 2 mL of Brain–Heart infusion (BHI) broth was poured into a test tube and vortexed. A 10 μL loopful of the sub-cultured growth of *A. baumannii* was inoculated into tubes. Further, a 10-μg ertapenem disk (Oxoid, Basingstoke, UK) was added to the suspension having the test isolate and incubated for 4 h at 35–37 °C. Just before the completion of 4 h incubation, 0.5 McFarland of *E. coli* ATCC 25922 was prepared and lawn cultured (by swabbing) on a MHA plate and left for 3–5 min to dry. Subsequently, the ertapenem disk was removed from the incubated test tube and placed on the MHA plate and kept in the incubator for 18–24 h [19]. The following interpretations were considered in the mCIM test: positive (6–15 mm inhibition zone), intermediate (16–18 mm), and negative (≥19 mm inhibition zone). The presence of small *E. coli* indicator strain colonies within the zone was considered positive [19]. For the eCIM experiment, two tubes (one for mCIM and the other for eCIM) containing 2 mL of BHI were prepared. A volume of 20 μL of 0.5 M EDTA was added into the second tube (for eCIM). Then, the procedure described above for the mCIM test was repeated with the test tubes. The interpretation of the test positivity was based on the zone of inhibition of *E. coli* ATCC 25922 in mCIM and eCIM plates. A ≥ 5 mm increase in zone diameter in the eCIM experiment as opposed to the respective mCIM plate indicated MBL production. A zone size ≤ 4 mm decrease (or no change in zone size) indicated a serine carbapenemase [19]. Known carbapenemase-producing *A. baumannii* organisms (OXA-23-like, OXA-40-like and OXA-58-like) were used as positive and negative controls.

Due to the very low sensitivity of the above methods, new modification of CIM, called simplified CIM or sCIM, was applied [20]. Briefly. *E. coli* ATCC 25922 was inoculated onto the MHA plate, following the routine disk diffusion procedure. Then, 1–3 overnight colonies of the test organisms were smeared onto an imipenem disk (10 µ) to allow one side of the disk to be evenly coated with the test bacteria; immediately afterward, the side of the disk having bacteria was placed on the MHA plate previously inoculated with *E. coli*. Plates were incubated at 35 °C for 16–18 h. The zone of inhibition around the disk shows a diameter of 6–20 mm or the satellite growth of indicator strain around the disk, indicated carbapenemase positivity; a zone of inhibition ≥ 26 mm was considered to be a negative result; a zone of inhibition of 23–25 mm was considered to be a carbapenemase-indeterminate result.

The ESBL phenotype was confirmed by means of the combined disk method, using antibiotic disks containing a combination of cephalosporin plus clavulanic acid, in conjunction with the corresponding cephalosporin disk alone, with the addition of cloxacillin in the medium (200 mg/L) to inhibit chromosomal AmpC β-lactamase, which can mask the synergistic effect with clavulanic acid. The following antibiotic disks were used: ceftazidime (30 μg), ceftazidime plus clavulanic acid (30/10 μg), ceftriaxone (30 μg), ceftriaxone plus clavulanic acid (30/10 μg) and cefotaxime (30 μg), and cefotaxime plus clavulanic acid (30/10 μg). Regardless of the zone diameters, an increase in zone diameter >5 mm for an antimicrobial agent tested in combination with clavulanic acid, in comparison with its zone size when tested alone, indicated probable ESBL [15].

### 2.3. Molecular Detection of Resistance Genes

Uniplex PCR was employed to detect genes encoding carbapenemases belonging to class A (*bla*_KPC_) [21] and class B MBLs genes (*bla*_VIM_, *bla*_IMP_, and *bla*_NDM_) [22,23,24]. Multiplex PCR was applied to identify class D β-lactamase genes (*bla*_OXA-23_, *bla_O_*_XA-24/40_, *bla*_OXA-51_, *bla*_OXA-58_, and *bla*_OXA-143_) [25,26]. The isolates positive for *bla*_OXA-23-like_ and *bla*_OXA-24-like_ genes in the multiplex assay were further analyzed using single primers for the respective genes. The genetic context of *bla*_OXA-51_ and *bl*a_OXA-23_ genes was determined by PCR mapping with primers for IS*Aba1* combined with forward and reverse primers for *bla*_OXA-51_ or *bl*a_OXA-23_ [27]. Under standard PCR conditions, amplification of different ESBL encoding genes, including PER [28], TEM [29], CTX-M [30] and SHV [31], were carried out according to the standard protocols.

### 2.4. Inter-Array Genotyping Kit CarbaResist

The genotyping of 10 randomly selected *A. baumannii* isolates (seven from UHCZ and three from UHCSM) was carried out with the microarray-based CarbaResist Genotyping Kit, in line with the manufacturer’s instructions, version 1012012100004 (INTER-ARRAY, fzmb GmbH, Bad Langensalza, Germany). Genomic DNA was extracted from monoclonal overnight cultures with the Qiagen DNeasy Blood and Tissue Kit. The unfragmented DNA was then amplified by one primer for each target sequence (antisense) and was internally labeled with biotin dUTP. The obtained ssDNA products were then transferred into the ArrayWells to perform the hybridization. The wells contained 230 probes for the distinct genes encoding the most relevant carbapenemases, ESBL and AmpC, aminoglycoside (*aac(3″)*, *aad1*, *aad1*, *aph*, *arm*, *rmt*) and fluoroquinolone resistance (*qnr* A, B, C, S). The wells were washed to remove any unbound DNA, and horseradish peroxidase (HRP)-conjugated streptavidin was bound to all of the hybridized sections, resulting in dark spots on the chip due to an enzymatic reaction. The detection of these spots and data acquisition was carried out automatically by the INTER-VISION Reader.

### 2.5. Whole-Genome Sequencing (WGS)

Five representative isolates (three from UHCZ and two from UHCSM) had their genomes sequenced as previously described [32]. Briefly, isolates were grown overnight on blood agar plates and had their DNA isolated using the DNeasy UltraClean Microbial Kit (Qiagen, Hilden, Germany). Sequencing libraries were prepared with the NEB Next^®^ Ultra™ II FS DNA Library Prep Kit for Illumina^®^ (New England Biolabs, Frankfurt, Germany). The libraries were sequenced, 2 × 250 bp paired end, in an Illumina MiSeq. The resulting Fasta files were assembled using the SKESA assembler (Version 2.4.0) as part of the MBioSEQ Ridom Typer software (Ridom GmbH, Münster, Germany, version 10.5.5). Genotyping was performed by a core-genome MLST (cgMLST), based on a core genome of 2390 target alleles (core) and 1083 target alleles (accessory), and was used to determine clonality. The 7-loci MLST sequence types were extracted from the assembled genome using PubMLST [33]. Antimicrobial resistance genes were identified using NCBI AMRFinderPlus.

An additional five strains were genotyped by conventional multilocus sequence typing according to the Oxford scheme. Seven housekeeping genes (*glt*A, *gyr*B, *gdh*B, *rec*A, *cpn*60, *gpi*, *rpo*D) were amplified and sequenced.

### 2.6. Conjugation

The conjugal transfer of meropenem resistance was carried out in mixed broth cultures at 35 °C. *E. coli* J65 resistant to sodium azide was used as the recipient [34]. Carbapenemase-producing transconjugants were selected on MacConkey agar containing meropenem (10 µg/L) and sodium azide (100 mg/L) to ensure that selected isolates were the recipient *E. coli* containing the transferred resistance gene. The frequency of conjugation was determined relative to the number of donor cells. The co-transfer of resistance to gentamicin, tetracycline, sulfamethoxazole–trimethoprim, chloramphenicol, and ciprofloxacin was determined in addition to β-lactam resistance transfer. Colonies growing on combined plates were subjected to identification by MALDI-TOF and, if confirmed to be *E. coli*, to antibiotic susceptibility testing and PCR for the detection carbapenemase-encoding genes.

### 2.7. Characterization of Plasmids

Plasmid incompatibility groups were determined by PCR-based replicon typing (PBRT) according to Bertini et al. [35]. Multiplex PCR was performed with primers specific for 18 incompatibility groups of plasmids found in *A. baumannii*.

## 3. Results

### 3.1. Bacterial Isolates and Patients

**Figure 1:**
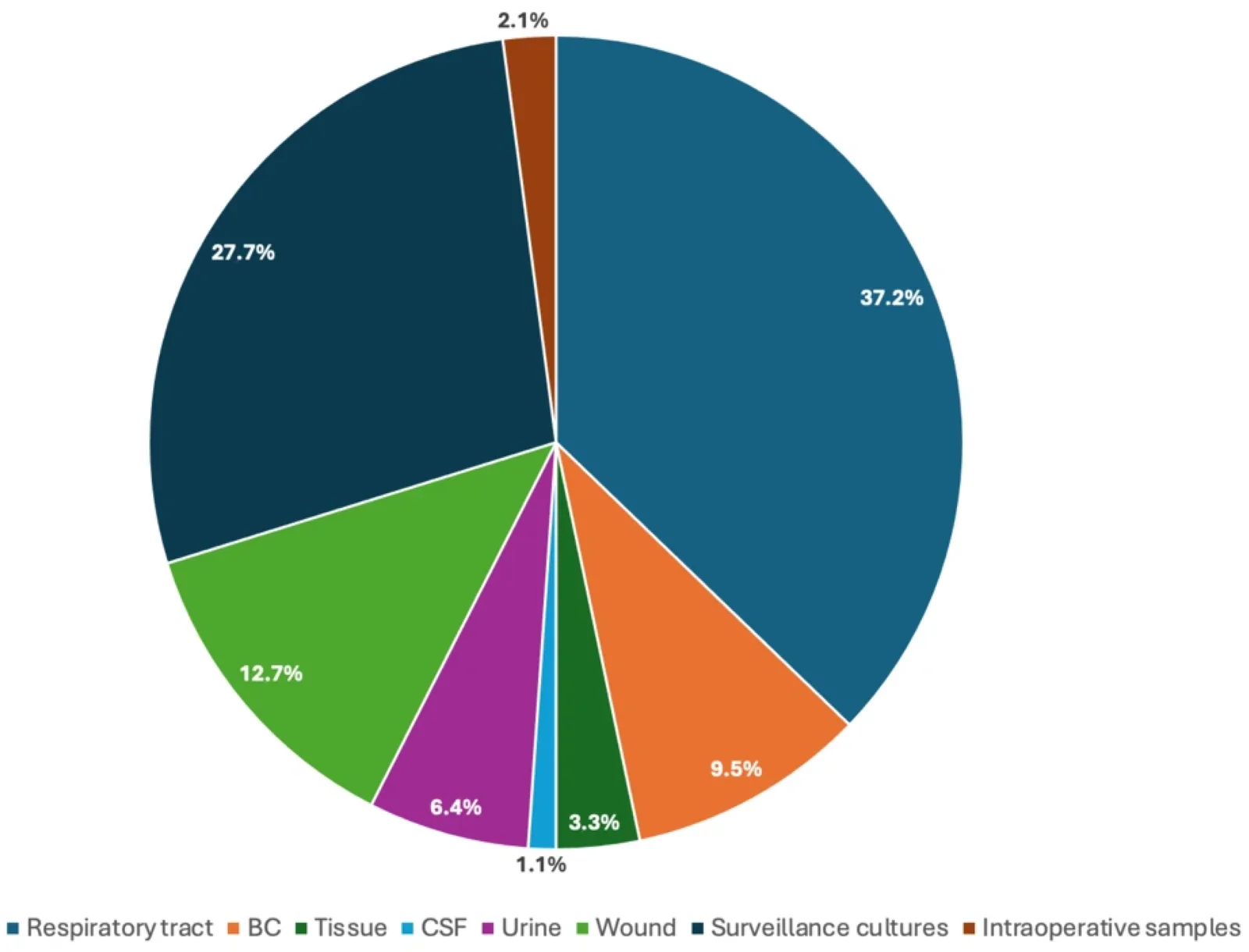
Distribution of the 94 isolates according to the specimen type. BC-blood culture; CSF-cerebrospina fluid.

In total, 94 non-copy (one per patient) *A. baumannii* isolates were collected from two hospital centers: UHCZ surgical ICUs (38) and UHCSM surgical ICUs (46 isolates). In addition, 10 outpatient isolates were also collected at the UHCZ. The majority of isolates originated from the respiratory tract (37%), followed by surveillance cultures (29%) (Figure 1). There were 67 males (71%) and 27 females (29%) included in the study. The age range was 19 to 93 years, with a median value of 65. A total of 37% of patients had an infection, and the others were only colonized with CRAB. Out of 84 hospitalized patients, 42% died (n = 35). Pneumonia was diagnosed in 23 patients (24%) and wound infection in 14 patients (15%). Urinary tract infection (UTI) was diagnosed in five patients (5%), mostly in the outpatient setting, associated with urinary catheters (catheter-associated urinary tract infection (CAUTI)). Colistin and ampicillin–sulbactam were the antibiotics used to treat CRAB infections in both hospitals. In UHCZ, a combination of both antibiotics was administered, while in UHCSM, patients received either colistin alone or ampicillin–sulbactam in monotherapy. Ten patients succumbed to septic shock and multiorgan failure (MOF). The Charlston comorbidity index for survival prediction varied between 0 and 11, with a median value of 5. The length of hospital stay ranged from 8 to 529 days (median 34) and ICU stay from 0 to 529 days (median 25). A particularly long ICU stay was observed in a patient with burn infection caused by CRAB (529 days). Forty-two out of 84 hospitalized patients (49%) had a CCI above four, indicating high mortality risk. A favorable therapeutic outcome was recorded in 61% of the patients harboring the OXA-23-positive strain and 50% with the OXA-24/40-producing strain. On the other hand, the median hospital and ICU stay was longer in the OXA-23-positive group compared to OXA-24/40 carriers (38 vs. 20 days and 24 vs. 12 days, respectively).

### 3.2. Antimicrobial Susceptibility and Phenotypic Tests for β-Lactamases

**Figure 2:**
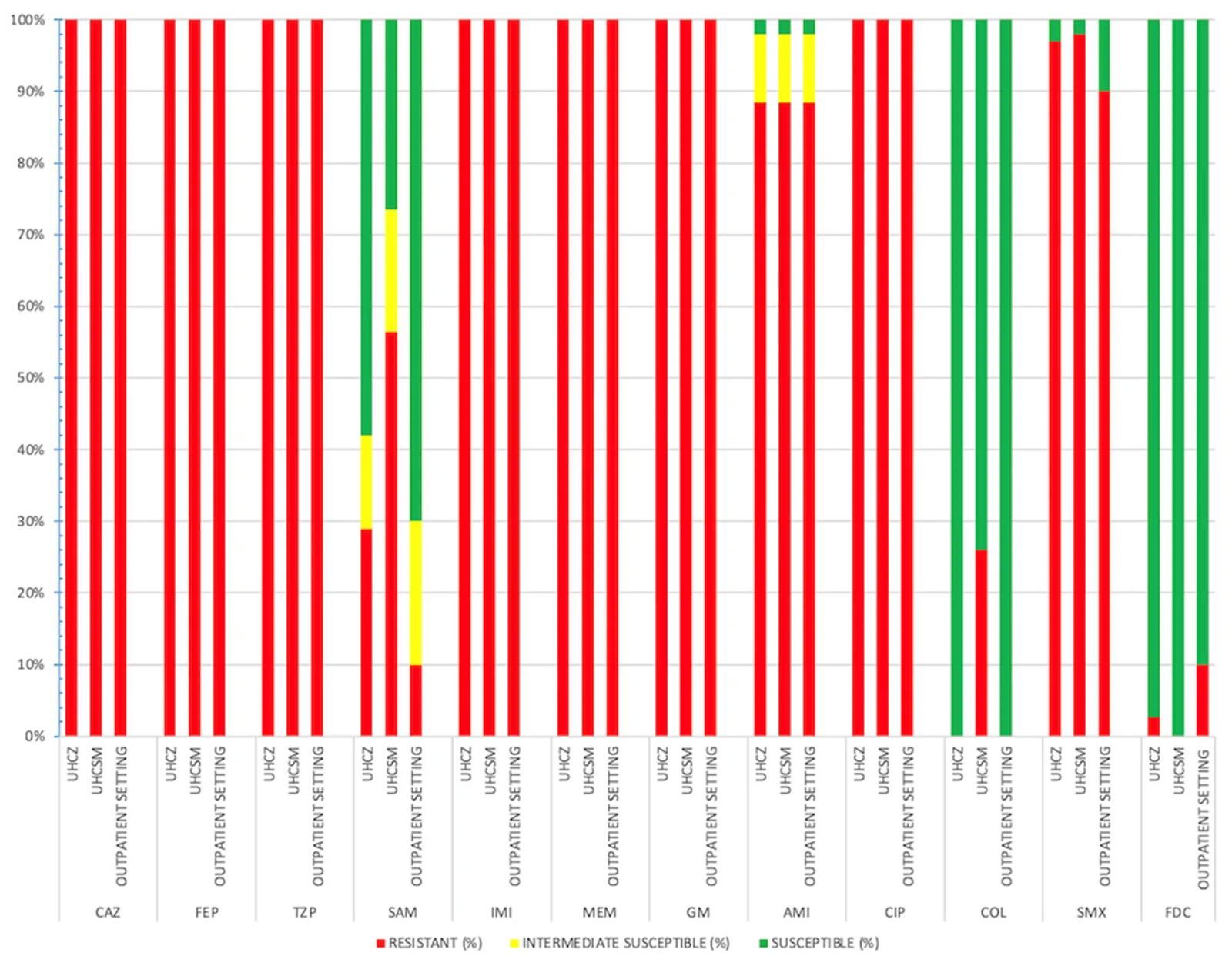
Antibiotic susceptibility of CRAB isolates according to the center: UHCZ with 38 isolates, UHCSM with 46 isolates and 10 from outpatient setting. **LEGEND**: Resistant, red; intermediate susceptible, yellow; susceptible, green. Abbreviations: CAZ—ceftazidime; FEP—cefepime; TZP—piperacillin–tazobactam; SAM—ampicillin–sulbactam; IMI—imipenem; MEM—meropenem; GM—gentamicin; AMI—amikacin; CIP—ciprofloxacin; COL—colistin; SMX—sulphamethoxazole–trimethoprim; FDC—cefiderocol; UHCZ—University Hospital Centre Zagreb; UHCSM—University Hospital Centre Sestre Milosrdnice.

All isolates were resistant to ceftazidime, cefepime, piperacillin–tazobactam, imipenem, meropenem, gentamicin and ciprofloxacin (Table 1). A high resistance rate was observed for amikacin—88% (n = 83). The overall rate of resistance to ampicillin–sulbactam was 41% (n = 39), but it was much higher among hospital isolates, with a rate of 45% (n = 38) vs. 1% (n = 1) among community isolates (Figure 2). Colistin resistance reached 12.7% (n = 12) and was detected only in UHCSM. According to DDST, the resistance rate to sulphamethoxazole–trimethoprim reached 97% (n = 91) and to levofloxacin 100% (n = 94), whereas cefiderocol remained active against 98% (n = 92) of the isolates. All isolates were CRAB and categorized as XDR, as only colistin and cefiderocol demonstrated consistent activity against the majority of isolates. Cloxacillin did not lower the MICs of either imipenem or meropenem, indicating no effect of AmpC on carbapenem susceptibility.

The MHT and CIM test exhibited poor sensitivity, with only 32% and 30% of isolates being identified as carbapenemase producers, respectively. The isolates exhibited weak positivity in MHT with very slight indentation of indicator *E. coli* strain growth. eCIM tested negative in all isolates, indicating the lack of an MBL. The new modification of CIM sCIM did not increase sensitivity and yielded only 28% (n = 27) positive results. All isolates tested negative for an ESBL, as there was no significant difference in the zone diameter between disks supplemented with clavulanic acid and control disks without inhibitor (−1–3 mm).

### 3.3. Molecular Detection of Resistance Genes

PCR identified *bla*_OXA-23-like_ as the dominant carbapenemase gene, with 71% of positive isolates (67/94). Moreover, it was the dominant gene in both hospitals (50%, n = 18 in UHCZ and 95%, n = 44 in UHSM), while in outpatient settings *bla*_OXA-24/40-like_ outnumbered *bla*_OXA-23-like_ genes (60%, n = 6). IS*Aba1* was detected upstream of *bla*_OXA-23_. No other acquired carbapenemase or ESBL genes were detected.

### 3.4. Inter-Array CarbaResist Kit Analysis

Out of ten analyzed isolates, there were six with *bla*_OXA-23-like_ and four with *bla*_OXA-40-like_ genes, respectively (Table 2). All except one isolate tested positive for *arm*A genes encoding pan-aminoglycoside resistance, while *aac(3′)Ia*, *aadA1* and *aadA2* were found in two isolates, respectively. Six isolates possessed *sul*1 and three *sul*2 genes for sulphonamide resistance. None of the isolates harbored genes for acquired fluoroquinolone, trimethoprim and colistin resistance, as shown in Table 2.

### 3.5. WGS

Of five isolates subjected to WGS, two harbored *bla*_OXA-23_ and three *bla*_OXA-72_ genes belonging to *bla*_OXA-24/40_ group as shown in Table 3. Regarding to aminoglycoside resistance genes, except *armA* which was detected by CarbaResist Kit, additional *ant*(3″)-IIa *aph*(3″)-Ib *aph*(6)-Id genes which encode phosphorylases were found in all isolates as shown in Table 3. Sulphonamide resistance was mediated by s*ul1* and s*ul2* genes. There were no acquired fluoroquinolone resistance genes, but mutations in *gyr*A and *par*C genes were identified.

### 3.6. Transfer of Resistance Determinants

Attempts to transfer meropenem resistance via conjugation were not successful.

### 3.7. Plasmid Characterization

The plasmids from OXA-23-positive isolates belonged to Inc group 2 and 6, encoding *aci2* and *aci6* replicase genes, respectively.

### 3.8. Genotyping

**Figure 3:**
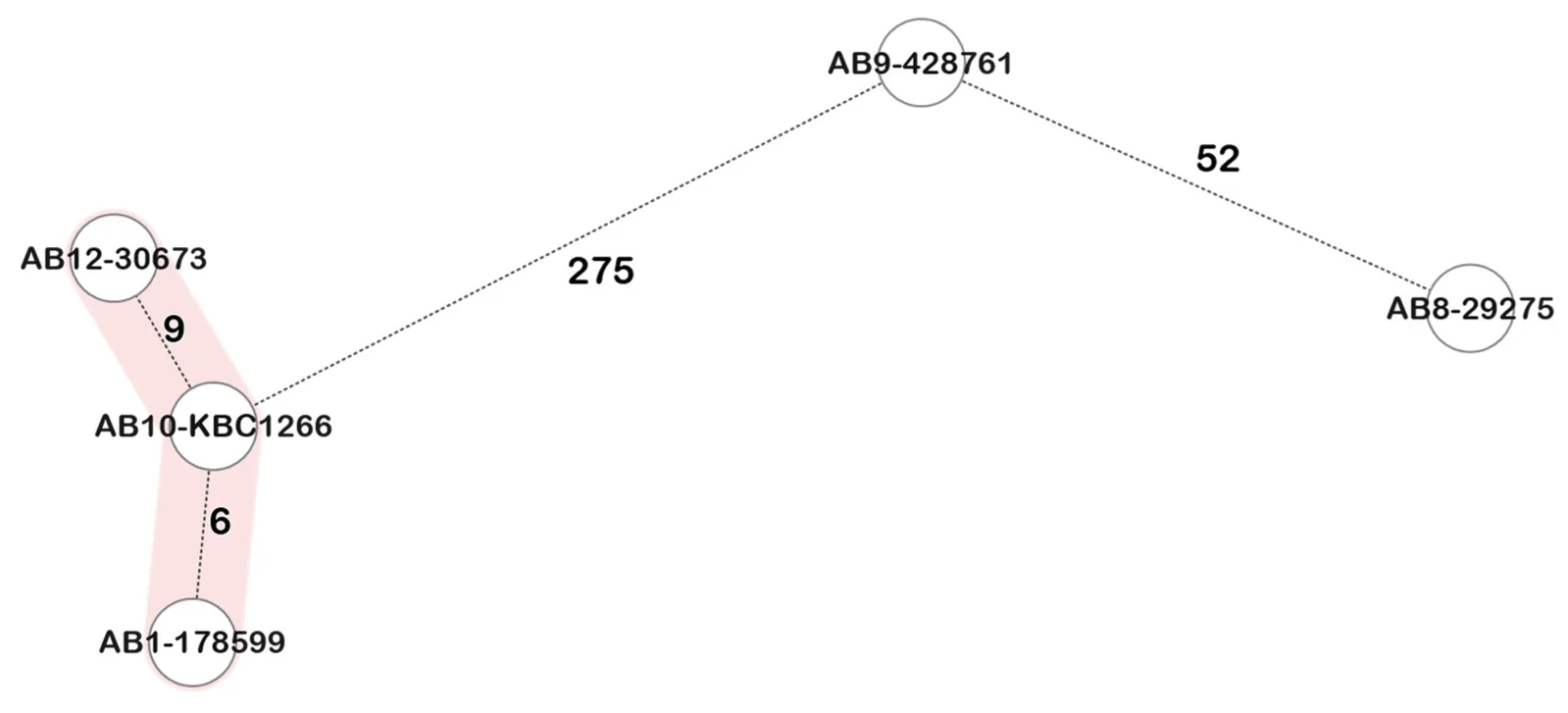
Minimum spanning tree of the 5 CRAB isolates using cgMLST based on 2390 target alleles. Isolate identifiers are shown in the nodes, and the number of different alleles is shown between the nodes. The transmission cluster 1 of the three OXA-72-producing isolates is illustrated by the shading around the nodes.

MLST retrieved from WGS revealed the existence of two clones among selected isolates: ST492 (a single-locus variant of ST2) carrying *bla*_OXA-72_ genes and ST2 harboring *bla*_OXA-23_ genes. ST2 and variants are also known as IC2. A phylogenetic tree of the five isolates demonstrated the existence of one transmission cluster containing three OXA-72 producing isolates (Figure 3). Two OXA-23-positive isolates did not cluster together and were considered singletons. MLST carried out on additional five strains identified ST208, an ST that corresponds to ST2 on the Pasteur scheme.

## 4. Discussion

In previous studies conducted during the COVID-19 pandemic, CRAB was mainly associated with VAP and BSI. However, in our present study there were also isolates obtained from wound infections (ischemic and diabetic ulcers), indicating lapses in hospital hygiene measures. Urinary isolates were linked to CAUTIs, attributable to biofilm production capacity. Infections caused by CRAB were associated with high mortality, reaching 42%, and prolonged hospital stay. The majority of patients had high CCI, indicating a lot of comorbidities, which is in line with the low virulence of *A. baumannii*, which, as a typical opportunistic pathogen, affects debilitated patients.

The study showed the switch from the predominance of OXA-24/40 in the pre-pandemic period [7,8] to the domination of OXA-23 after the pandemic. In the multicenter study carried out in the period from 2009 to 2011, there was an equal number of OXA-23-, OXA-24/40- and OXA-58-producing isolates [8]. However, in the present study, OXA-58 completely dissapeared, and we have the absolute predominance of OXA-23, which could be attributed to better hydrolysis capacity and a higher level of resistance on one side, plasmid location of *bla*_OXA-23_ enabling horizontal spread of the genes on the other side, or clonal expansion of OXA-23 strains. To answer this, typing of more isolates will be required. Interestingly, of the five isolates we sequenced, it was the OXA-72 isolates that showed the most clonality.

Croatia belongs to the countries with a very high rate of CRAB, reaching around 95% in practically all institutions [36], similar to the majority of Southeastern European countries like Bulgaria, Romania and Greece [37].

The studies carried out during the COVID-19 pandemic showed the dissemination of both OXA-23-like and OXA-24/40-like carbapenemases in hospitals providing care for COVID-19 patients in Zagreb [12,13]. It seems that OXA-23-like and OXA-24-like clones are endemic over the last decade in Balkan countries, including Croatia, Serbia and Bosnia and Herzegovina [38,39,40,41]. Among isolates positive for *bla*_OXA-40-like_ genes, *bla*_OXA-72_ was, similarly as in the previous studies, the only allelic variant. It is hard to explain the long-lasting predominance of this specific allelic variant in Balkan countries.

ESBLs were not found, which is in line with the rare occurrence of these resistance determinant among this species. There are only sporadic reports, describing mostly SHV, CTX-M and PER variants outside Europe [42,43]. CTX-M-115 was associated with ST78 *A baumannii* with OXA-72 and was associated with Russia in 2012 and Moldova in 2023 [44].

Interestingly, MBLs were never found among CRAB from Croatia, although they are increasingly reported in developing countries [45,46,47], but only sporadically found in Europe, usually associated with imported cases [48,49] in addition to a focal point in Serbia [41].

Resistance to aminoglycosides was due to a combination of *arm*A genes encoding 16S rRNA methylase and *aph*(3) and *aph*(6*″*) genes generating aminoglycoside modifying phosphotransferases. *Sul1* and *sul2* genes mediated sulphonamide resistance. There were no acquired fluoroquinolone resistance genes, but quinolone resistance mutations of chromosomal *gyr*A and *par*C genes were found. Antibiotic resistance genes content was strikingly similar in all tested isolates.

The presence of CRAB in surveillance cultures like rectal swabs and upper respiratory tract swabs indicated colonization and presents a silent reservoir for endogenous infections and vertical transfer within the hospital and to the community setting.

The study found weak agreement between phenotypic tests and PCR. Poor performance of phenotypic tests could be due to low efficacy of CHDL in general. Low catalytic power demonstrated in low Vmax despite high Km is a possible explanation for the failure of phenotypic tests. Some studies found CARBA NP is a better indicator for carbapenemase production in *A. baumannii* than the MHT and mCIM/eCIM test. Some authors have suggested use of a modified CIM method with longer incubation time (6 h) [50] and the addition of Triton to increase the permeability of bacterial cells to release the carbapenemases [51]. However, this could cause loss of specificity due to spontaneous hydrolysis of carbapenems. Other studies have found higher sensitivity of MHT, ranging from 70 to 80% [52,53]. On the other hand, mCIM was confirmed to have very low sensitivity by other authors, with around 30–35% of PCR-positive isolates being recognized as carbapenemase-positive [51,52,53]. For that reason, a new modification called simple CIM (sCIM) was recently implemented, which has a sensitivity of 80% compared to PCR [54].

In contrast to *Enterobacterales*, identification of carbapenemase positivity and type of enzyme does not affect antibiotic choice and has no clinical relevance. Recently, a new immunochromatographic test has been developed to detect the most important carbapenemases in CRAB [55]. However, unlike the similar test for *Enterobacterales*, it is not widely used in routine diagnostics in Croatia. Molecular and phenotypic analysis has no clinical impact, unlike in *Enterobacterales*, because the therapy is always based on antibiograms. Molecular analysis is important for epidemiological reasons, to track the dynamic changes in antibiotic resistance traits. In *Acinetobacter*, the choice of antibiotic is not affected by the type of carbapenemase, as there are currently no inhibitors of *Acinetobacter*-associated carbapenemases.

Outside hospitals, CRAB was previously reported in a nursing home in Zagreb [10], and hospital-associated isolates have been found in wastewater treatment plants [56]. This study reports diffusion of CRAB into the community setting among patients not hospitalized within last three months.

Plasmids belonging to groups 2 and 6 were previously shown to carry *bla*_OXA-23_ and *bla*_OXA-58_ genes [35]. However, due to the failure of meropenem resistance transfer and lack of transconjugant strains, we could not prove the plasmid origin of *bla*_OXA-23_ genes. Plasmid analysis is an important tool to analyze molecular epidemiology of the isolates, as we can track the horizontal transmission of plasmids into different strains and lineages.

Our CRAB isolates showed considerable concurrent resistance to fluoroquinolones and aminoglycosides, limiting the therapeutic options. We demonstrated an increase in colistin and ampicillin–sulbactam resistance rates compared to our previous studies. Such isolates pose a serious therapeutic problem because of their XDR phenotype. Resistance to colistin found in the minority of the isolates as a last resort antibiotic for CRAB infections is worrisome. However, cefiderocol could be considered a promising therapeutic option for CRAB infections, particularly for those strains that are resistant to colistin and other agents. Sulbactam, either alone or in combination, is increasingly recognized as a valuable therapeutic option for CRAB infections. However, in our study high resistance rates were observed.

ST2 was previously identified in Croatia, also in conjunction with *bla*_OXA-23_ genes [8]. ST492, a single-locus variant of ST2, was reported among pediatric isolates in Croatia and positive for *bla*_OXA-24-like_ [9] and recently in the neighboring country Serbia, also carrying the OXA-72-coding gene [41]. ST208 is reported in Croatia for the first time. In the past, OXA-23-positive organisms mainly belonged to widespread clone ST195 [57]. ST2, found among our OXA-23-positive isolates, belongs to, by far, the most common causes of CRAB outbreaks in intensive care units globally [11]. Similarly, as in other studies our OXA-23-positive organisms harbored the *armA* gene for pan-aminoglycoside resistance. 

New molecular methods like WGS enable detection of the whole resistome. The test identified additional aminoglycoside resistance genes like *ant(3′)-IIa aph(3″)-Ib aph(6)-Id*, which encode phosphorylases responsible for modification of aminoglycosides, which were not found by the Inter-array CarbaResist Kit.

The limitation of the study is the fact that WGS was done on a limited number of isolates, and thus it was not possible to prove clonal spread within hospital units involved in the study. We did not measure the effect of porin loss or upregulation of efflux pumps on antibiotic resistance. Moreover, we could not determine the effect of carbapenem resistance on the outcome, mortality rate and duration of hospital stay because all isolates were CRAB and there were no susceptible isolates for comparison.

Earlier studies were focused on hospital isolates, and in this study, community-acquired isolates were included. However, although the patients were not hospitalized in the last three months, there is usually a link with the hospital, such as attending a day-care service or diagnostic visits.

## 5. Conclusions

CRAB belongs to the WHO critical group of pathogens with very limited therapeutic options. Its ability to rapidly acquire antibiotic resistance has made it a challenging “superbug” to treat. This bacterium has evolved to rapidly adapt and overcome the challenges posed by a wide range of antibiotics. The relentless rise of antibiotic resistance has precipitated a global health crisis, with CRAB emerging as a particularly formidable pathogen. Colistin and cefiderocol were the only antibiotics showing consistent activity against these isolates.

In conclusion, the study demonstrated dissemination of the same CRAB clones, before and during the COVID-19 pandemic, with similar phenotypes and resistance determinants. Given the XDR phenotype and the observed clinical impact, the problem demands immediate response, including CRAB surveillance, infection control measures and antibiotic stewardship.

## Figures and Tables

**Table 1:** Minimum inhibitory concentrations (µg/mL) of various antibiotics against CRAB isolates.

Antibiotic	MIC Range	MIC_50_	MIC_90_	Number and % of Resistant Isolates
piperacillin–tazobactam	32- ≥ 128	128	≥128	94/94 (100%)
ampicillin–sulbactam	4- ≥ 128	128	≥128	39/94 (41%)
ceftazidime	64- ≥ 128	≥128	≥128	94/94 (100%)
cefepime	32- ≥ 128	≥128	≥128	94/94 (100%)
imipenem	16- ≥ 128	128	≥128	94/94 (100%)
meropenem	32- ≥ 128	≥128	≥128	94/94 (100%)
gentamicin	32- ≥ 128	≥128	≥128	94/94 (100%)
amikacin	16- ≥ 128	≥128	≥128	83/94 (88%)
ciprofloxacin	8- ≥ 128	≥128	≥128	94/94 (100%)
colistin	1–128	1	8	12/94 (13%)

**Table 2:** Inter-array CarbaResist Kit analysis of selected 10 isolates and Oxford scheme sequence types (STs).

Isolate and Protocol No	Res. Phenotype	β-Lactam	Aminoglycoside	Sulphonamide	ST
AB1 178599 UHCZ—outpatient	TZP, CAZ, FEP, IMI, MEM, GM, AMI, CIP	*bla*_OXA-40-like_	*armA*	*sul*2	ST208
AB2 370512 UHCSM	TZP, CAZ, FEP, IMI, MEM, GM, AMI, CIP	*bla*_OXA-23-like_	*armA*	*sul*1	ST208
AB8 29275 UHCSM	TZP, CAZ, FEP, IMI, MEM, GM, AMI, CIP	*bla*_OXA-23-like_	*armA*	*sul*1	ST208
AB9 428761 UHCZ	TZP, CAZ, FEP, IMI, MEM, GM, AMI, CIP	*bla*_OXA-23-like_	*armA*	*sul*1	ST208
AB10 1266 UHCZ	TZP, CAZ, FEP, IMI, MEM, GM, AMI, CIP	*bla*_OXA-40-like_	*armA*	*sul*2	ST208
AB11 11810 UHCZ	TZP, CAZ, FEP, IMI, MEM, GM, AMI, CIP	*bla*_OXA-40-like_	*armA*	*sul*2	ST208
AB14 175936 UHCZ	TZP, CAZ, FEP, IMI, MEM, GM, AMI, CIP	*bla*_OXA-40-like_	*aac(3′)Ia* *aadA1* *aadA2*	*sul*2	
AB23 UHCSM	TZP, CAZ, FEP, IMI, MEM, GM, AMI, CIP	*bla*_OXA-23-like_	*armA* *aphA*		
AB25 UHCZ	TZP, CAZ, FEP, IMI, MEM, GM, AMI, CIP	*bla*_OXA-23-like_	*armA* *aac(3’)Ia* *aadA1* *aadA2*	*sul*1	
AB28 100320 UHCZ	TZP, CAZ, FEP, IMI, MEM, GM, AMI, CIP	*bla*_OXA-23-like_	*armA*	*sul*1	ST208

**Table 3:** WGS of selected isolates showing resistance gene content, their Pasteur and Oxford MLST and cgMLST clustering; O—Oxford scheme, P—Pasteur scheme.

Isolate Number	β-Lactam	Aminoglycoside	Sulphonamide	Quinolone	Quaternary Ammonium	ST	Cluster
AB1 178599 UHCZ— outpatient	*bla*_OXA-72_ *bla*_OXA-66_ *bla*_ADC-30_	*armA ant(3″)-IIa aph(3″)-Ib aph(6)-Id*	*sul*2	GyrA_S81L ParC_S84L		ST492(P) ST208 (O)	1
AB8 29275 UHCSM	*bla*_OXA-23_ *bla*_OXA-66_ *bla*_ADC-30_	*armA ant(3″)-IIa aph(3″)-Ib aph(6)-Id*	*sul*1	GyrA_S81L ParC_S84L	*qacEdelta1*	ST2 (P) ST195 (O)	S
AB9 428761 UHCZ	*bla*_OXA-23_ *bla*_OXA-66_ *bla*_ADC-30_	*armA ant(3″)-IIa aph(3″)-Ib aph(6)-Id*	*sul*1	GyrA_S81L ParC_S84L	*qacEdelta1*	ST2 (P) ST208 (O)	S
AB10 1266 UHCZ	*bla*_OXA-72_ *bla*_OXA-66_ *bla*_ADC-30_	*armA ant(3″)-IIa aph(3″)-Ib aph(6)-Id*	*sul*2	GyrA_S81L ParC_S84L		ST492 (P) ST208 (O)	1
AB12 30673 UHCSM	*bla*_OXA-72_ *bla*_OXA-66_ *bla*_ADC-30_	*armA ant(3″)-IIa aph(3″)-Ib aph(6)-Id*	*sul*2	GyrA_S81L ParC_S84L		ST492 (P) ST208 (O)	1

## Data Availability

The original contributions presented in the study are included in the article. Further inquiries can be directed to the corresponding author. The data are not publicly available due to privacy and ethical restrictions. The raw sequencing reads generated in this project were submitted to the European Nucleotide Archive under the study accession number PRJEB112739.

## Abbreviations

CRAB—carbapenem-resistant *Acinetobacter baumannii*; ESBL—extended-spectrum β-lactamases, ESC—expanded-spectrum cephalosporins; CHDL—carbapenem-hydrolyzing class D oxacillinases; MIC—minimum inhibitory concentration; WGS—whole-genome sequencing, BSI—bloodstream infection; CLSI—Clinical Laboratory Standard Institution; EUCAST—European Committee for Antimicrobial Susceptibility Testing; MHA—Mueller–Hinton agar; OKNV—OXA-48, KPC, NDM, VIM; CAZ—ceftazidime; FEP—cefepime; CTX—cefotaxime; CRO—ceftriaxone; AMT—aztreonam; IMI—imipenem, MEM—meropenem; WHO—World Health Organization; ST—sequence type; ICU-intensive care unit.
